# Pilot randomised controlled trial of protective socks against usual care to reduce skin tears in high risk people “STOPCUTS”: study protocol

**DOI:** 10.1186/s40814-015-0005-3

**Published:** 2015-04-01

**Authors:** Roy J Powell, Christopher J Hayward, Caroline L Snelgrove, Kathleen Polverino, Linda Park, Rohan Chauhan, Philip H Evans, Rachel Byford, Carolyn Charman, Christopher J W Foy, Andrew Kingsley

**Affiliations:** 1 Research and Development Directorate, Noy Scott House, Royal Devon and Exeter NHS Foundation Trust, Barrack Road, EX2 5DW Exeter, Devon UK; 2 Peninsula Clinical Trials Unit at Plymouth University (PenCTU), ITTC Building, Plymouth Science Park, PL6 8BX Plymouth, Devon UK; 3 University of Exeter Medical School, St Luke’s Campus, Magdalen Road, EX1 2 LU Exeter, Devon UK; 4 NIHR Clinical Research Network, South West Peninsula, Noy Scott House, Royal Devon and Exeter NHS Foundation Trust, Barrack Road, EX2 5DW Exeter, Devon UK; 5 Research and Development Office, Leadom House, Gloucester Royal Hospital, GL1 3NN Gloucester, Gloucestershire UK; 6 Northern, Eastern and Western Devon Clinical Commissioning Group, County Hall, Topsham Road, EX2 4QD Exeter, Devon UK

**Keywords:** Skin tears, Pre-tibial lacerations, Prevention, Protective socks, Dermatuff

## Abstract

**Background:**

Skin tears are traumatic injuries occurring mostly on the extremities due to shearing and friction forces that separate the epidermis and the dermis from underlying tissues. They are common and occur mostly in older adults and those taking medications that compromise skin integrity. Pretibial skin tears can develop into leg ulcers, which require lengthy, expensive treatment to heal. Traumatic injuries are the second most common type of wounds after pressure ulcers in care homes and are the commonest reason for older adults to require the attention of a community nurse. Common causes of skin tear injuries are bumping into furniture and other obstacles, using mobility aids, transfer to/from wheelchairs, getting in and out of bed and falls. No effective preventative measures currently exist but knee-length, protective socks are now available that contain impact-resistant Kevlar fibres (of the type used in stab-proof vests) and cushioning layers underneath.

**Methods/design:**

In this pilot parallel group, randomised controlled trial, 90 people at risk of skin-tear injury will be randomised with equal allocation to receive the intervention or usual care. They will be recruited from care homes and from the community via general practices and a research volunteer database. Pilot outcomes include recruitment, eligibility, attrition, ascertainment of injuries and completion of outcome measures. Acceptability of the intervention and of study participation will be explored using semi-structured interviews. The proposed primary outcome for the future definitive trial is skin tear-free days. Secondary outcomes are skin tear severity, health status, specific skin-tears quality of life, capability and fear of falling, measured at baseline and the end of the study and in the event of a skin tear.

**Discussion:**

The results of this study will be used to inform the development and design of a future randomised controlled trial to assess the effectiveness and cost-effectiveness of a unique and innovative approach to skin tear prevention.

Approval was granted by the NRES - Cornwall and Plymouth Research Ethics Committee (13/SW/013). Dissemination will include publication of quantitative and qualitative findings, and experience of public involvement in peer-reviewed journals.

**Trial registration:**

Current Controlled Trials: ISRCTN96565376

## Background

Skin tears are traumatic wounds involving a piece of skin of varying size being peeled away from underlying tissues either completely or leaving a partial or intact skin flap. They often occur as a result of rubbing, an abrasion or a glancing blow to an arm or leg (i.e. from a fall or being struck or poked obliquely). The most commonly cited definition of skin tear is that of Payne and Martin: “A skin tear is a traumatic injury occurring principally on the extremities of older adults as a result of shearing or friction forces which separate the epidermis from the dermis (partial thickness wound) or which separate both the epidermis and the dermis from underlying structures (full-thickness wound)” [1]. These are common injuries [2-6]. Some prevalence data comes from America [7-10] and more recently from Japan where point prevalence was 3.9% amongst 410 patients in long-term care [11] and Australia where two elderly care rehabilitation units reported 10% [12]. A non-systematic review [9] reported skin tear incidence between 2.23% and 41.5% and prevalence between 6.6% and 23.5% in US care homes. In Pennsylvania, skin tear reporting became mandatory for healthcare facilities in 2004 [13], where 88.2% of the 2,807 skin tears were in patients aged over 65 years. Fourteen percent of an American 120-bed nursing home population sustained a skin tear per month with an average of 2.67 tears per resident [14]. A recent wound point prevalence audit undertaken in North Devon [15] in 16 care homes revealed 195 wounds amongst 115 of 458 residents (25%). Traumatic injuries were the second most common wound type (37, 19%) after pressure ulcers (87, 45%).

Several changes occur in the skin that increase its susceptibility to traumatic injury [16,17]. These changes are due to intrinsic ageing and cumulative extrinsic factors such as photoageing and polypharmacy. They include vascular atrophy and deterioration of the dermis as collagen and elastin fibres become more sparse and disordered, holding the skin layers together less tightly [18]. Older patients may have also taken oral steroids that compromise skin integrity and tensile strength and cause wounds to heal more slowly [19-22]. They may also be less aware that an injury has occurred due to decreased pain perception and tactile sensitivity, including diabetic neuropathy [23]. Fragile skin is most common in people aged over 75 years. There were 10 million people in this age group in the UK in 2012, estimated to increase to 17 million in 2032 [24]. Skin tears are unpleasant and provoke anxiety. They can take a long time to heal and are prone to infections. Whilst arm injuries are more common, leg injuries may develop into leg ulcers, which may require lengthy, expensive treatment [25]. Typical causes of injury leading to skin tears include wheelchair use, bumping into obstacles, transfers and falls. There are best practice guidelines for treatment and preventing infections and ulcers [26]. Prevention includes staff education, regular assessment, ensuring clothing does not rub, removing obstacles and moisturising the skin [9,27,28].

The novel, knee-length protective socks have a leg section woven from Kevlar [29] and elasticated nylon using the “terry sandwich” method. This gives a flat, slightly ribbed and stretchy, outer woven base which provides a tough, cut and abrasion-resistant exterior. There is a mesh of loops on the inside to provide a cushioning and impact resistant inner layer. The stretchiness is sufficient to fit a range of leg diameters within each size without applying excess pressure, and the socks are held up with a light elastane soft top band. The foot of the socks is manufactured from cotton as laceration protection is not usually required for the feet. Compression hosiery could be worn underneath if required as the socks are not designed to offer any compression themselves. Patients requiring such hosiery are excluded from this study, however, as this may confound any protective effects.

Apart from small-scale, uncontrolled testing during development, there has been no trial of the effectiveness and cost-effectiveness of this unique and innovative approach to skin tear prevention. This study will test the feasibility of running such a trial by addressing areas of uncertainty within the study protocol.

## Method/design

**Figure 1:**
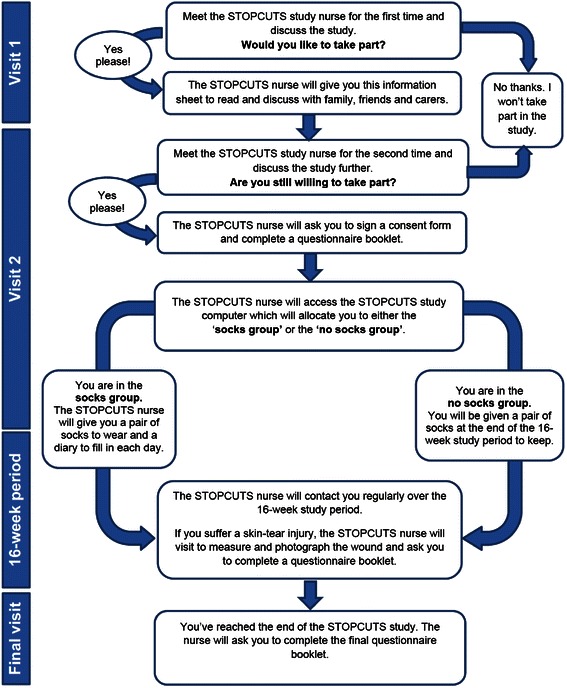
Participant pathway diagram (taken from the Participant Information Sheet).

The study is an open, parallel group, randomised controlled study in which participants are randomised to one of two groups; the intervention group will wear the protective socks on a daily basis for a period of 16 weeks whilst the participants in the control group will wear their usual clothing. See Figure 1 for the patient flow diagram and Table 1 for the study schedule. The study is conducted in Devon, UK and aims to recruit 90 participants, 45 in each arm. The main eligibility criteria for participants are presented in Table 2. Experiences of using the socks and/or taking part in the study will be captured through semi-structured interviews with a purposive sample of participants from both the intervention and control arms of the study.

**Table 1:** Tabulated study schedule

Study procedure	Set up (Visit 1)	Baseline/Study Day 0 (Visit 2)	Study Day 1 (the day after consent)	Upon new injury	Weekly* for 16 weeks	Study Day 112	Qualitative data collection
Identify potentially eligible participants	X						
Provide Participant Information Sheet	X						
Informed consent		X					
Collection of baseline information		X					
EQ5D-5 L questionnaire		X		X^c^		X	
ICECAP-O questionnaire		X		X^c^		X	
FES-I (short) questionnaire		X		X^c^		X	
Randomisation		X					
Provision of socks (intervention arm only)		X					
Wearing of socks (intervention arm only)			X --------------------------------- > X				
Completion of participant diary (intervention arm only)			X --------------------------------- > X				
Photograph wound				X^b^			
Measurement of wound				X^b^			
Wound scoring				X^b^			
Cardiff wound impact schedule^a^				X^c^		X^d^	
Adverse event recording			X --------------------------------- > X^e^				
Research nurse to visit care home for progress checks					X		
Patient interviews							X
Focus groups							X

^*^Regular contact (visit or telephone), approximately weekly.

^a^Completed only by participants who experienced one or more skin tear injuries during their study participation.

^b^Performed within one working day following injury.

^c^Performed seven days post-injury.

^d^Completed on Day 112 by all participants who experienced skin tear injury during the study. In addition, participants who incur an injury during week 16 (Day 106 to 112 inclusive), will also complete the questionnaire seven days post-injury.

^e^Day 112 or Day 112 + 1 to 7 days if a skin tear injury occurs during week 16.

**Table 2:** Main eligibility criteria (inclusion and exclusion criteria)

Inclusion criteria	Exclusion criteria
Resident in a care home or living in the community and at high risk of skin tears.	Not competent to give informed consent in the opinion of the recruiting nurse.
Aged 65 years or older.	Bedbound.
Ambulatory and/or wheelchair user able to take part in daily activities within a care home or in the community.	Being treated for current lacerations or ulcers on their legs.
	Participant likely to use graduated compression stockings or similar during the intervention period.

The participants will be recruited primarily from Care Quality Commission registered care homes. Residents of care homes are widely recognised to be an under-researched population and at high risk of suffering skin tears. Depending on recruitment rate of both homes and participants, up to 45 homes are expected to take part. In order to augment recruitment, patients in the community will also be invited to participate. For logistical reasons, in order to maximise efficiency in terms of research nurse resource, recruitment efforts will be focused on three geographical areas successively (Exeter, Exmouth/Sidmouth and Mid Devon, roughly representing urban, coastal and rural situations, respectively).

### Research question

The research question for the eventual definitive trial will be “Are protective, Kevlar-woven socks (‘Dermatuff’) when compared with care as usual, effective and cost-effective in providing lower leg skin protection in people at risk of suffering skin tears in terms of their incidence and severity?” The research question for this pilot is: “Is it feasible to conduct a trial of ‘Dermatuff’ protective socks compared to care as usual in people at risk of suffering skin tears?”

### Trial objectives

This pilot study is intended to pave the way for a definitive randomised controlled trial assessing the effectiveness and cost-effectiveness of the new socks in protecting the lower legs. The aim of this pilot study is to provide the necessary information for the planning of the future trial. It will test the feasibility of running such a trial and provide estimates of recruitment (care homes and residents, and patients in primary care), trial attrition, completion of questionnaire-based quality of life measures, baseline scores and standard deviations of those measures in this population. It will test the feasibility of gathering data on skin tears occurring in care homes and in the community by research nurses. It will assess the acceptability of using the protective socks and also of participating in the trial. It will provide useful information about the distribution of the variables to inform the sample size calculation for a larger trial. It will also provide an estimate of the impact of any contamination (during subsequent interviews).

Specific objectives of this pilot study are therefore:

- To examine the feasibility and acceptability of the research design, methods and outcome measures prior to a definitive randomised trial.
- To assess the processes for capturing outcome data.
- To refine the intervention if appropriate through qualitative work on acceptability.
- To determine recruitment and retention rates (at care home and resident or community participant level) to a randomised trial.
- To estimate concordance with the intervention.
- To estimate attrition rates from the study.
- To consider outcome measures which are important and appropriate for participants for use in the full trial.
- To evaluate the appropriateness of two systems of skin tear classification as outcome measures for the main trial.
- To estimate rates of questionnaire completion.
- To obtain baseline estimates of scores on the proposed outcome measures in this clinical population.
- To estimate the variability of outcomes to inform the sample size of the definitive trial.
- To calculate the sample size required for a definitive trial.
- To help establish the eligibility criteria for the future definitive trial (primary care patients, care homes and residents).
- To estimate the ability to obtain cost and effectiveness measures.
- To refine the clinical protocol for the use of protective socks in the full trial.
- To gain further understanding of the practicalities of running a trial in this population.
- To obtain feedback from nursing homes regarding the acceptability of the study.

### Randomisation and blinding

Randomisation will be achieved by means of a Web-based system created by the Peninsula Clinical Trials Unit in conjunction with the study statistician. The participants will be allocated to the protective socks intervention or to standard care in equal proportions, using blocks of fixed size to generate the allocation sequence and achieve balance in the numbers of participants allocated to each group. Variable block sizes were considered but would provide no advantage since the number of participants recruited at each care home is likely to be small. Randomisation will be stratified by geographical area (i.e. Exeter, Exmouth/Sidmouth and Mid Devon).

The Web-based randomisation system created and maintained by the clinical trials unit will allow secure access via computers or other devices with Internet connectivity. Research nurses will be provided with log-in details and will perform randomisation from the care home by smart phone. The system ensures allocation is concealed prior to performing randomisation. Telephone randomisation will be available during working hours (09:00 to 17:00, Monday to Friday inclusive) in the unlikely event the Web-based service is unavailable.

Blinding will not be possible for participants or research nurses. Blinding is possible for the data analyst, however, by coding the group allocation in the data file.

### Sample size

The target sample size for this pilot trial is 90 participants. The sample size was based on a group sample of 40 with a 20% inflation to allow for dropout and loss to follow-up. A sample of 40 in each group was considered to be adequate for providing robust estimates of skin tear incidence, skin tear-free days and standard deviations for quality of life and other outcome measures.

### Recruitment

#### Identification of care homes and approach

Care homes will be identified from Care Quality Commission lists with the advice of the local Tissue Viability Service. The homes will be sent a study brochure to invite them to express an interest in taking part. Staff from interested homes will be invited to a study event at which the study protocol will be explained in a group setting. All homes expressing interest will be visited by a research nurse to explain the study in more detail and confirm willingness to take part. All care homes that take part will be required to sign a letter of agreement to acknowledge their understanding of what their involvement entails. At this set-up visit, the research nurse will provide study-specific training for the staff and will also identify potentially eligible residents.

The research nurses will not provide any clinical management; their role is primarily to establish contact with potential participants and manage the collection of study data. If, however, during the conduct of the study, the research nurse notices any condition not related to the study which, in their professional opinion requires attention, they have a duty of care to report this to the care home manager.

#### Recruitment of care home residents

Potentially eligible residents will be identified to the research nurse by care home staff at the home’s set-up visit (Visit 1). The research nurse will approach potential participants, explain the study, show the resident a sample of the protective socks and leave written information (i.e. the Research Ethics Committee (REC) approved Participant Information Sheet). Care home staff will be provided with a site file containing essential documents and contact details (mobile telephone numbers and email addresses) for the research nurse.

#### Informed consent

The research nurse will return within a day or two (ensuring residents have had sufficient time to read, understand and discuss the Participant Information Sheet) to answer questions and ask whether the potential participants would like to take part in the study. The research nurse will be responsible for adjudging the residents’ capacity to provide informed consent, and this will be achieved through discussion with the resident to determine whether or not the study information has been retained and understood. The research nurse will consider the guidance in the Mental Capacity Act 2005 Code of Practice when assessing mental capacity. Those who do wish to take part will be asked to give their formal consent by signing the consent form. The written informed consent process will be undertaken by the research nurse who will be trained in Good Clinical Practice and the informed consent process.

#### Recruitment of patients from the community

Study entry criteria remain the same for this group of participants except that they do not need to be resident in a care home. Time points for data collection and the outcome data items collected will be the same.

#### Participant identification via Primary Care

The Clinical Research Network (South West Peninsula) will support the identification and recruitment of research active general practices (GPs) into the study. Practices that decide to participate will be contacted by the research nurse to discuss the requirements of the study and the search criteria. GP staff will search for patients aged 65 years or older who have used oral steroids for more than a month in the prior 12 month period. Lists generated from the search will be screened for suitability by a doctor at each participating practice and unsuitable patients excluded, e.g. those with terminal illness, mental illness etc. A member of the practice staff will send suitable patients an invitation letter on GP headed paper, including a study reply form and reply-paid envelope. Patients are asked to return the reply form to the research team if interested in receiving more information about the study. Patients that respond to say that they are interested in receiving more information will be contacted by a member of the research team and an appointment made for the research nurses to meet with the patient. The GP may send one follow-up letter to all the patients originally invited with a sentence advising that the letter should be ignored if they have already responded. Patients who return the reply form to the research team will then undergo the recruitment process described below.

#### Participant identification via the NIHR Exeter Clinical Research Facility

Potential participants may also be identified via the “Exeter 10000” research volunteer bank managed by the NIHR Exeter Clinical Research Facility (Exeter CRF). The volunteer bank database will be searched by authorised members of the Facility for volunteers aged 65 years and older who have used oral steroids for more than a month in the prior 12-month period. A member of the facility staff will send suitable volunteers an invitation letter on Exeter CRF headed paper, including a study reply form and reply-paid envelope. Volunteers will be asked to return the letter (including the reply form) to the Exeter 10000 team who will forward their name to the research team if interested in receiving more information about the study. Volunteers who return the reply form will then undergo the recruitment process described below.

#### Community recruitment process

On receipt of a completed reply form, a research nurse will telephone the patient or volunteer using the contact details provided on the reply form, once the person has had at least 24 h to read the study information. The research nurse will ascertain potential eligibility by explaining the full study entry criteria to the person. If the research nurse is satisfied that the person is potentially eligible, and willing to take part, the research nurse will arrange a visit at which eligibility will be confirmed and informed consent obtained. Visits may be performed at persons’ homes or at a local community hospital or GP surgery if mutually convenient.

#### Informed consent, randomisation and baseline assessment of community participants

At this initial visit, the research nurse will explain the study and demonstrate a pair of the protective socks. The written informed consent process will be undertaken by a research nurse trained in GCP and the informed consent process. Having reconfirmed eligibility, those who do wish to take part will be asked to give their formal consent by signing the consent form. Baseline measures and randomisation will then be performed as per protocol. On successful completion of this visit, the REC approved GP letter will be posted to the participant’s GP, if the participant consents to their GP being informed.

#### Participant retention

Plans to promote participant retention and complete the follow-up include encouragement from the research nurses who will be in regular telephone and face-to-face contact with the participants, whether resident in a care home or living independently in the community.

### Intervention group

The protective socks are manufactured by Dermatuff Limited. The socks are CE marked and are registered with the Medicines and Healthcare products Regulatory Authority (MHRA) as “Skin Tears Protection System Wear”, a Class I Medical Device. Materials used in the manufacture of the socks are in conformity with all relevant and required standards including the ISO 10993 series evaluating biocompatibility of a medical device prior to a clinical study.

The research nurse will carry a stock of protective socks and, before leaving the care home, the research nurse will visit those randomised to wear protective socks to provide socks of the correct size. The socks are available in a choice of grey or beige colour. The choice of colour will be recorded by the research nurse. Three pairs of socks will be provided to the care home for each participant in the intervention group. The participants will be able to keep these socks at the end of their participation in the study.

The participants in the intervention group will be asked to wear the protective socks during their waking hours every day for a period of 16 weeks, starting from the morning following the day they sign consent, which will be designated as “Day 1.” Care home staff will be asked to encourage participants to wear the socks and to assist them to put the socks on, if necessary. However, it will be emphasised that if participants become unwilling to wear the socks or want to take them off, they will be free to make that choice. The participants should continue to wear their normal footwear during participation in the study.

#### Participant diary

The participants in the intervention group will be given the first of 16 weekly diaries at this visit and will be asked by the research nurse to complete the diary on a daily basis. The participants will be asked to use the diary to record the extent to which they wear the socks each day and the reasons for removing them (if applicable), plus any negative or positive comments about wearing the socks. A new weekly diary will be provided to the participant each week.

### Control group

The participants in the control group will be managed as usual. This includes any routine procedures to reduce the risk of lacerations, but otherwise they will wear their normal clothing. At the end of their participation, each participant from the control group will be offered one pair of socks to keep for their own use.

### Outcome measures

The measures proposed for a future definitive trial will be collected in this pilot study. The primary outcome of a future trial will focus on skin-tear free days and the incidence and severity of skin tears.

#### Procedure in the event of a skin tear injury

Care home staff or the community participant themselves will be asked to contact the research nurse by telephone immediately on becoming aware of an injury (i.e. as soon as possible on the day of the injury or the following morning in the case of an overnight injury). The research nurse will visit the resident or patient as soon as practicable (within 1 to 2 working days) of being contacted in order to collect the following:

- Date and approximate time of injury.
- Brief description of injury cause.
- Type of lower leg clothing and footwear worn at the time of injury (including whether protective socks were in place for participants in the intervention group).
- Size of injury (length, width and total area using a grid tracing method).
- Category of injury using the Payne-Martin and STAR classifications (as described in Appendix).
- A photograph of the injury.
- Information relating to the initial management of the injury (dressing type, referral to external healthcare professional such as GP, district nurse and hospital emergency department).

The research nurse will inspect the wound for signs of infection and will also check that the injury has been noted in the care home records (in the case of care home participants). Injured participants will complete outcome measure questionnaires 7 days post-injury.

### Primary outcome

A useful way to express the primary outcome measure is the number of skin tear-free days. Each participant will enter the study with no unhealed skin tear injuries and will remain in the study (unless withdrawn prematurely) for 112 days (i.e. 16 full weeks). Therefore, each participant has the potential to experience 112 skin tear-free days. In the event of a skin tear injury, the number of days from the date of injury until the date it is healed will be subtracted from 112 to give the number of skin tear-free days. A healed injury is defined as one which has an absence of scab and full epithelial covering that does not require continuance of dressing for absorption of exudate (sometimes a dressing may be left on a healed but delicate wound for a few days after healing). The date of healing will be identified by the visiting research nurse via wound inspection during regular, approximately weekly, visits and in collaboration with care home staff as necessary. Participants who experience a skin tear injury in week 16, or who have an injury discovered at week 16, will continue to provide follow-up information beyond that date and skin tear-free days will be expressed as a proportion of the total follow-up time.

### Secondary outcomes

#### Skin tear assessments

There is no universally accepted classification system for the assessment of skin tears. Payne and Martin developed the first classification system in 1990, and this was updated in 1993 [1]. Problems associated with inter-rater reliability testing of the Payne and Martin classification system, and its poor utility in Australia, led to a study that resulted in the Skin Tear Audit Research (STAR) Classification System. The STAR Classification System is commonly used in Australia, with evidence of implementation reported within the UK [30].

In the main trial, skin lacerations will be measured in centimetres (length, breadth and area) and graded either according to Payne-Martin categories or the STAR Skin Tear Classification System. The photographs will inform an assessment of the reliability of the reported grading of the injury and will be performed by a tissue viability nurse specialist blinded to allocation.

Skin tear assessments will be performed upon learning of an injury, within 24 h if possible.

#### Other secondary outcome measures

- Standardised measure of health status (EQ-5D-5L): collected at baseline, 16 weeks and in the event of a skin tear injury.
- Assessment of capability (ICECAP-O): collected at baseline, 16 weeks and in the event of a skin tear injury.
- Assessment of fear of falling (Short FES-I): Collected at baseline, 16 weeks and in the event of a skin tear injury.
- Disease-specific quality of life measured by Cardiff Wound Impact Schedule: Collected in the event of a skin tear injury and at 16 weeks (from participants who have had a skin tear).
- Adverse events caused by the socks.
- Health care resource use: collected in the event of a skin tear injury.
- Outcome measures for the pilot study are:
- Recruitment rate for homes.
- Proportion of participants (home residents) eligible.
- Recruitment rate for participants.
- Attrition and loss to follow up.
- Ascertainment of injuries.
- Completion and completeness of study questionnaires.
- Estimates of the distribution of outcome measures.
- Feasibility of the workload.
- Acceptability of the intervention to participants.
- Acceptability of study participation to participants.

### Withdrawal and dropout

The participants may withdraw their consent at any time. The participants will be advised that they do not have to provide a reason for withdrawing their consent but if they are willing to provide a reason when asked by the research nurse the reason will be recorded. Standard care will not be affected by a participant’s decision to withdraw from the study. Data collected prior to withdrawal will be included in the study analysis unless a participant specifically requests that their data are removed from the database. If a participant is adjudged to have lost mental capacity during the 16-week study period, that participant will be withdrawn from the study. Data already collected with consent will be retained and used in the study. No further data will be collected or any other study procedures carried out on or in relation to the participant. It is anticipated that some participants allocated to the socks group may discontinue wearing the socks but will still be happy to fill in the outcome measures at the end of 16 weeks and in the event of a skin tear.

### Adverse and serious adverse events

In the STOPCUTS study, adverse events which are not serious and not related to study participation will not be recorded or reported. Adverse events which are serious and/or related to study procedures/intervention will be recorded. Multiple symptoms should be recorded as separate events. The events will be reported to the Chief Investigator, Sponsor and the Peninsula Clinical Trials Unit on a designated report form which will capture the research nurse’s opinion on the relatedness of the event to study procedures/intervention, and also on the expectedness of the event. Adverse reactions to the socks are expected to be uncommon. The following list of potential symptoms will be used as a reference when assessing the expectedness of adverse device effects.

- Allergic-type skin reaction.
- Miliaria.
- Chafing.
- Excessive sweating under the socks.
- Skin tears or bruising caused by putting on or removing the socks.
- Pain, discomfort, numbness, swelling or any other condition caused by socks which are too tight.
- Falls or other accidents caused by slipping or tripping as a result of wearing the socks.

Accumulative summaries of adverse reactions will be reviewed periodically by the Trial Steering Committee.

### Data handling

#### Participant numbering

Each participant will be allocated a unique four-digit identification number. Each participant will be identified in all study-related documentation by his/her study number and initials. A record of names linked to participants’ study numbers will be maintained by the research nurse and stored securely for administrative purposes.

#### Data capture and transfer

Data will be recorded on study-specific case report forms (CRFs) by the research nurse and directly into questionnaire booklets and diaries by participants. Photographs taken by research nurses will be saved, transferred and stored according to the Royal Devon and Exeter Hospital’s local policy. The images will be stored securely on the hospital system in accordance with data protection regulations.

#### Data entry

Completed paper CRF’s will be checked and signed by the research nurse before being sent to the clinical trials unit. Original paper CRF pages, questionnaire booklets and diaries will be posted to the trials unit at agreed time points for double-data entry onto a password-protected database in accordance with the trials unit standard operating procedures (SOP’s). Research nurses will also have the option of entering CRF data at agreed time points directly onto the study database via the study website. These data will not be double entered. Forms will be tracked using a Web-based study management system. Double-entered data will be compared for discrepancies using a stored procedure. Discrepant data will be verified using the original paper data sheets.

#### Data confidentiality

The research nurses will ensure that participants’ anonymity is maintained on all documents and images. Data will be collected and stored in accordance with the Data Protection Act, 1998. Within the trials unit, anonymised paper-based study data will be stored in locked filing cabinets within a locked office. Electronic records will be stored in a Structured Query Language (SQL) server database, stored on a restricted access, secure server maintained by Plymouth University. The website will be encrypted using Secure Sockets Layer data encryption (SSL). Direct access to the study data will be restricted to members of the study team, the Sponsor and the Peninsula Clinical Trials Unit. Access to the database will be overseen by the trials unit data manager and trial manager. Copies of study data retained at study sites (i.e. care homes) will be securely stored for the duration of the study prior to archiving.

#### Archiving

Following completion of study data analysis, the sponsor will be responsible for archiving the study data and essential documentation in a secure location for a minimum period of 5 years after the end of the study. No study-related records should be destroyed unless or until the sponsor gives authorisation to do so.

### Statistical methods

The study will be analysed using the statistical package SPSS v. 20 (IBM Corp, New York). Missing data will be investigated and the proportion missing will be recorded but are expected to be minimal. Multiple imputation methods or any specific imputation methods recommended by the authors of the questionnaire will be used to obtain realistic estimates of scores for future planning of questionnaire usefulness. All the questionnaires concerning acceptability of the socks will be scored and summarised using appropriate measures of central tendency and dispersion. Data on lacerations will also be summarised in a similar way. Agreement on wound grading between research nurses and a blinded tissue viability expert (AK) will be assessed from anonymised photographs using weighted kappa. Numbers of eligible residents, recruitment, attrition and loss to follow-up (as per the Consolidated Standards of Reporting Trials (CONSORT) diagram) will be reported as proportions, confidence intervals will be reported wherever appropriate. The main analysis will describe skin tear-free days and the incidence and severity of skin lacerations in each group.

The pilot study will determine the feasibility of how the outcomes can be measured. These will be used to inform the sample size of the full-scale randomised controlled trial. In that trial, we intend to compare the incidence of skin damage between the two groups on an intention-to-treat basis and the size of any wounds by objective assessors (research nurses). The clustering effects of homes will be an issue in the analysis of a future definitive randomised controlled trial, and sample size inflation will need to be considered for a mixed effects model with “homes” as a random effect.

#### Economic evaluation

The economic evaluation in the future definitive trial will estimate the additional NHS cost per quality-adjusted life years (QALY) gained by the use of these protective socks. QALY estimation will be based on the EuroQol descriptive system (EQ-5D-5L) collected at baseline and at 16 weeks for all participants, and at 7 days after a participant incurs a skin tear injury to the leg. In the pilot study, a note will be made of how many of the participants have difficulties understanding or completing EQ-5D-5L to assess whether there is a need to include proxy assessment in the design of the main trial.

The study will collect data on the resources used by the care homes, GPs or healthcare professionals and the NHS in the management of skin tears. The primary source of these data will be the participants’ medical records of their normal procedures, visits from district or tissue viability nurses, visits to and from GP’s and any care needs arising from adverse events. The pilot study will assess whether care home records and or participants’ medical notes are adequate to describe resource use in a costable format.

#### Qualitative interviews and focus groups

Semi-structured qualitative interviews with ten participants from each arm of the study will be conducted at the end of the follow-up period by an independent researcher. These will include a range of participants with varying degrees of mobility across the areas. Experiences of using the protective socks, their acceptability and/or taking part in the study will be captured. The participants will be selected using purposive sampling informed by research nurse data on their clinical progress with regard to skin tears, perceived protection from knocks and falls, withdrawals, adverse events and any problems with the skin-tear and/or questionnaire data collection process. Interviews will be digitally recorded and transcribed with the main themes identified using content analysis.

Two focus groups will be held to explore the usefulness of the protective socks. These will include professionals from the Tissue Viability Service and care home staff with experience of participants assigned to the intervention arm of the study. Holding two focus groups provides easy access for staff of care homes across the large geographical area of the pilot trial.

### Quality control

The research nurse will check completed case report forms for missing data or obvious errors before the forms are sent to the trials unit. Data will be monitored centrally for quality and completeness, and every effort will be made to recover data from incomplete forms where possible. The data manager at the trials unit will oversee data tracking and data entry and initiate processes to resolve data queries where necessary, and the trial manager will devise a monitoring plan specific to the study which will include both central monitoring strategies and study site visits if appropriate.

Participating care homes will be required to permit the trial manager or deputy or representative of the sponsor to undertake study-related monitoring to ensure compliance with the approved study protocol and applicable standard operating procedures, providing direct access to source data and documents as requested. All study procedures will be conducted in compliance with the protocol and according to the principles of the International Conference on Harmonisation Good Clinical Practice (ICH GCP). Procedures specifically conducted by the clinical trials unit (e.g. randomisation, data management, study management and study monitoring) will be conducted in compliance with the trials unit’s standard operating procedures.

A trial management team meets regularly to discuss the progress of the trial and address any issues that arise. A Trial Steering Committee, with an independent chair, independent clinician and independent statistician meets approximately every 6 months to oversee the conduct and safety of the trial.

### Ethics

The study will be performed subject to approval by the Cornwall and Plymouth NRES Research Ethics Committee, including any provisions of Site Specific Assessment (SSA) and local Research and Development approval.

## Discussion

Skin tears are the second most prevalent injury type in care homes after pressure ulcers [15] and a cause of considerable pain, misery and lost confidence in older people and those with fragile skin due to long-term use of medications such as steroids. They also represent a considerable cost to the NHS. Existing measures to prevent skin tears have limited effectiveness in this susceptible population [27,28]. This pilot trial and any future definitive trial arising from it will focus on this important area of healthcare. This pilot will address the uncertainties in planning a future definitive randomised controlled trial.

The areas of uncertainty to be addressed are typical in a study of this nature, the feasibility of recruitment (of care homes, general practices and of individual participants), the suitability of outcome measure assessments and their timing and the distribution of variables (and therefore the number of participants needed in a full definitive trial of effectiveness). In particular, it will focus on the acceptability of the socks. Preliminary work and early patient and public involvement indicated that they might not be aesthetically pleasing to participants because of their current limited colour range (charcoal grey or tan) and perceived thickness. Doubts have also been raised about comfort and fitting as there are currently only four sizes available (small, medium, medium-wide and large). Other sizes and colours could be manufactured in the future. We also don’t know whether the socks can be worn and tolerated during the different seasons of the year. We hope to tease these issues out in the qualitative interviews and the daily diaries.

One possible weakness of the pilot study design is the potential for contamination because the socks are commercially available on the Internet. Participants and/or their relatives or carers will know of the existence of Dermatuff socks, having read about them in the study Participant Information Sheet. It would not be difficult to obtain some socks for themselves if they have originally been assigned to the control group and decide that they need some. In primary care recruitment, it is also possible that GPs may recommend Dermatuff socks to their patients in the control group, hence contaminating the sample. Any contamination that occurs in this pilot will be recorded, and the information will be used in the planning of a future trial. Cluster randomisation would not prevent this direct contamination, but it might minimise the “indirect contamination” caused by control group participants seeing intervention group participants wearing the socks, and altering their own behaviour to better protect their legs — by perhaps being more careful how they move around or taking other protective measures — such as wearing their own leg-wear more often.

A particular strength of this trial is that it is taking place partly in care homes, which represent an under-researched population of older people. This is thought to be a difficult area for researchers to tackle because of perceived problems with access to residents and the prevalence of dementia in this population, which limits the number eligible to take part in research. Sadly, older people with dementia are at even greater risk of skin tears than those without it [8]. Furthermore, care home representatives involved in the planning of this trial have informed us that people with dementia are much more at risk of receiving skin tears. The Mental Capacity Act 2005 states that for people lacking capacity to consent, any research that they may be permitted to take part in must be connected with an impairing condition affecting such a person or its treatment. “Impairing condition” means a condition which is (or may be) attributable to, or which causes or contributes to (or may cause or contribute to), the impairment of, or disturbance in the functioning of, the mind or brain. It could be argued that increased skin tear prevalence amongst people with dementia is attributable to the condition, and that any efforts to reduce their incidence should be pursued. If this were the case, given the high prevalence of dementia in care homes, a future trial might be conducted entirely within the care home environment with appropriate consultation with carers, as the Mental Capacity Act 2005 requires.

Dermatuff socks offer hope of some protection from skin tears when very few other measures have worked, and this pilot will go some way to indicating whether it is worth carrying out a full scale definitive trial in the future to determine their effectiveness and cost effectiveness.

## Trial status

The STOPCUTS trial has been designed as a pilot, parallel group randomised controlled trial of effectiveness. The initiation took place at the Royal Devon and Exeter Hospital, Devon, UK in July 2013 after approval by the research ethics committee with two subsequent amendments to refine the recruitment process. The trial commenced in July 2013. Recruitment is ongoing and is scheduled to end in October 2014. Data collection is scheduled to continue until February 2015 and results will be analysed in July 2015.

## Appendix

### Skin tear categorisation

#### Payne-Martin classification

- Category I: Skin tear without loss of tissue. The epidermal flap either completely covers the dermis or covers the dermis to within 1 mm of the wound margin
  - o Sub category Ia: Linear type
  - o Sub category Ib: Flap type
- Category II: Skin tear with partial tissue loss
  - o Sub category IIa: Scant tissue loss (25% or less)
  - o Sub category IIb: Moderate to large loss of tissue (>25% loss of the epidermal flap)
- Category III: Skin tear with complete tissue loss.

#### STAR Skin Tear Classification System

- Category 1a: A skin tear where the edges can be realigned to the normal anatomical position (without undue stretching) and the skin or flap colour is not pale, dusky or darkened.
- Category 1b: A skin tear where the edges can be realigned to the normal anatomical position (without undue stretching) and the skin or flap colour is pale, dusky or darkened.
- Category 2a: A skin tear where the edges cannot be realigned to the normal anatomical position and the skin or flap colour is not pale, dusky or darkened.
- Category 2b: A skin tear where the edges cannot be realigned to the normal anatomical position and the skin or flap colour is pale, dusky or darkened.
- Category 3: A skin tear where the skin flap is completely absent.
